# Multifactorial Determinants of Cardiopulmonary Resuscitation Quality in the Emergency Department: A Narrative Review

**DOI:** 10.7759/cureus.107369

**Published:** 2026-04-19

**Authors:** Chinnam Vishnupriya, Anagani Hrushikesh, Annet Maria Thomas, Gireesh Kumar

**Affiliations:** 1 Emergency Medicine, Amrita Institute of Medical Sciences, Kochi, IND

**Keywords:** cardiopulmonary resuscitation, cpr quality, emergency department, human factors, in-hospital cardiac arrest

## Abstract

In-hospital cardiac arrest survival rates remain 20-30% despite guideline advances, with emergency department (ED) resuscitation uniquely challenged by crowding, staff turnover, and diagnostic uncertainty. While technical CPR benchmarks are well-defined, consistent ED performance varies widely, suggesting system, human, and environmental factors significantly influence outcomes. Successful CPR outcomes depend not only on the quality and technique of resuscitation being performed but also on various contributing factors that concurrently influence the overall effectiveness of CPR in the patient. This narrative review synthesizes multifactorial determinants of CPR quality specific to ED practice.

PRISMA 2020-guided narrative synthesis across PubMed, Scopus, and Web of Science (January 2010-March 2026). Boolean search terms identified 1,247 records; two independent reviewers screened 1,189 titles/abstracts and assessed 189 full texts, excluding 159 (non-ED focus n = 102, pediatric/out-of-hospital n = 34, and non-English n = 23), yielding 52 studies categorized into five thematic domains.

ED crowding independently reduces chest compression fraction (CCF) 11% and delays defibrillation 42 seconds (OR 1.4); leadership absence causes 2.1-fold longer shock delivery; procedural interruptions from intubation and point-of-care ultrasound (POCUS) average 12-19 seconds reducing CCF 8-17%; intraosseous access achieves 92% success vs. 59% IV failure; mechanical CPR devices improve CCF 92% vs. 78% manual but show no survival benefit; family presence adds 4.7-second pauses despite psychological benefit to 85% families.

ED CPR quality reflects a complex interplay of system strain (CCF reductions 11-28%), human factors (leadership delays OR 2.1), technology integration challenges (POCUS pauses 12-19 seconds), procedural workflow, and patient complexity. Current quality improvement must extend beyond technical training to address these modifiable ED-specific barriers.

## Introduction and background

In-hospital cardiac arrest (IHCA) remains a significant contributor to hospital mortality and is frequently regarded as an indicator of institutional resuscitation efficacy. Contemporary registry data suggest that more than 290,000 adults experience IHCA annually in the United States, with survival to hospital discharge typically reported between 20% and 30%, depending on patient characteristics and arrest circumstances [1,2]. Although hospitals have observed incremental improvements over the past decade, they still have uneven survival rates. Importantly, this variation persists even after adjustment for case mix, suggesting that differences in systems of care and institutional practices contribute to outcome disparities [1,3].

Outcomes also differ according to the location of arrest within the hospital. Survival patterns in intensive care units, monitored wards, and emergency departments (EDs) are not identical, reflecting differences in monitoring intensity, staffing models, and response systems [4,5]. Variability in rapid response activation, documentation practices, and physiological surveillance further contributes to heterogeneity in reported results [2,6]. It is well recognized that abnormal vital signs often precede cardiac arrest, yet early warning signs may not always be identified or acted upon promptly [6]. These observations underscore that cardiac arrest outcomes are shaped not only by the quality of CPR itself but also by the broader clinical environment in which deterioration occurs.

The ED represents a particularly complex setting for resuscitation. The ED is different from intensive care units because the number of patients, the accuracy of diagnoses, and the number of interruptions are constantly changing. Crowding remains a persistent challenge and has been associated with delays in critical interventions and higher mortality among severely ill patients [7,8]. Evidence also suggests that overcrowding adversely affects outcomes following IHCA through delayed defibrillation (42 seconds longer), reduced chest compression fraction (CCF) (11% lower), and impaired team coordination [9]. As the interface between prehospital services and inpatient care, the ED is characterized by rapid patient turnover, multiple handoffs, and evolving team composition [10]. In such an environment, maintaining consistent adherence to evidence-based resuscitation practices proves particularly challenging.

Recent American Heart Association (AHA) 2025 and European Resuscitation Council (ERC) 2025 guidelines emphasize early defibrillation, CCF >80%, compression depth 5-6 cm at 100-120/minute with full recoil, ventilation 8-10/minute, and physiological targets including end-tidal carbon dioxide (ETCO₂) >10 mmHg and diastolic blood pressure >25 mmHg [11,12]. Observational studies connect these objective measures of CPR quality to better survival rates. Real-time feedback devices and ETCO₂ monitoring improve adherence to compression targets and cut down on peri-shock pauses by 30%. Despite these advances, achieving consistent performance in ED practice remains challenging due to human factors, system design, and patient complexity [13-19].

Technical competence alone does not determine resuscitation quality. Human factors leadership clarity, structured communication, situational awareness, and cognitive load management under stress prove central to effective cardiac arrest management [20-25]. The ED environment, with its competing demands and frequent interruptions, amplifies cognitive burden during resuscitation [26]. These pressures, along with the design of the system and the reliability of the technology, affect how closely teams can follow the suggested performance standards.

Patient characteristics introduce additional complexity. Advanced age, frailty, multiple comorbidities, and pre-arrest physiological instability affect both immediate resuscitation success and longer-term neurological recovery [6,27,28]. Decisions regarding initiation or termination of resuscitation must align with goals of care, adding ethical complexity to high-risk clinical situations [29]. Disparities in access to monitoring tools, staffing resources, and critical care infrastructure contribute to outcome differences across healthcare systems [30].

Although researchers have examined individual components of cardiac arrest management extensively, fewer analyses consider how these elements interact specifically within the ED. The ED creates a unique setting where system-level pressures, human performance dynamics, technological variability, patient heterogeneity, and ethical considerations all occur simultaneously. Understanding CPR quality in this context requires consideration beyond technical performance alone.

In this narrative review, we examine the multifactorial determinants influencing CPR quality in the ED and synthesize current evidence into five clinically meaningful domains that may guide targeted quality improvement initiatives.

## Review

Methods

A systematic literature search was performed in PubMed/MEDLINE, Scopus, and Web of Science (January 2010-March 2026), used as the primary reference source, in order to track down the suitable articles in the English language with pertinent significance regarding factors affecting the CPR quality, utilizing Boolean strings: ("in-hospital cardiac arrest" OR IHCA OR "emergency department" OR ED) AND ("CPR quality" OR "chest compression" OR "resuscitation performance" OR "team dynamics" OR leadership OR "human factors" OR technology OR capnography OR "feedback device" OR ethics OR documentation).

Two independent reviewers screened the records identified through the database search in accordance with the PRISMA 2020 principles. Out of the total 1,247 articles, screening was performed based on the title and abstract. During full-text assessment, the irrelevant articles were excluded because they did not focus on ED resuscitation, were limited to pediatric or out-of-hospital settings, case reports, conference abstracts, or were not available in the English language. The final evidence synthesis included 52 studies, comprising peer-reviewed original research studies, systematic reviews, narrative reviews, and international resuscitation guidelines from the AHA and the ERC relevant to determinants of cardiopulmonary resuscitation quality and associated performance metrics in the ED. There was no risk of bias in compliance with the narrative design or quantitative synthesis. A structured summary table (Table 1) of the primary principle studies included in this review was prepared, detailing the author, year, country, study design, sample size, and principal findings to enhance transparency of evidence contributing to the thematic synthesis. No formal quality assessment was conducted because of the variability of narrative designs, although high-impact guidelines and registries were given precedence.

**Table 1:** Summary of principal studies included in the narrative review on determinants of CPR quality in the emergency department CPR - cardiopulmonary resuscitation; ED - emergency department; IHCA - in-hospital cardiac arrest; OHCA - out-of-hospital cardiac arrest; POCUS - point-of-care ultrasound

Author/Year	Country	Study Design	Sample Size	Key Findings
Girotra et al. [1], 2012	USA	Observational registry study	84,625 IHCA patients	Survival after IHCA improved over time, highlighting the impact of better resuscitation systems.
Merchant et al. [3], 2012	USA	Observational multicentre study	135 hospitals	Significant inter-hospital variation in IHCA rates suggests system-level factors influence outcomes.
Perman et al. [4], 2016	USA	Registry analysis	151,071 IHCA patients	Arrest location significantly affected survival outcomes, with monitored settings showing better survival.
Chan et al. [5], 2016	USA	Nationwide survey	131 hospitals	Hospitals with structured resuscitation practices had higher survival rates.
Andersen et al. [6], 2016	USA	Retrospective cohort	44,531 patients	Abnormal vital signs prior to arrest were common, emphasizing the role of early detection systems.
Sun et al. [8], 2013	USA	Retrospective cohort	995,379 admissions	Emergency department crowding adversely affected admitted patient outcomes.
Jun et al. [9], 2025	South Korea	Retrospective observational study	786,702 ED visits	ED overcrowding increased the occurrence of in-hospital cardiac arrest.
Yeung et al. [17], 2014	UK	Randomized simulation trial	67 teams	Prompt and feedback devices improved chest compression quality during CPR.
Bobrow et al. [18], 2013	USA	Prospective interventional study	5,000+ OHCA cases	Scenario-based training with audiovisual feedback improved CPR quality and survival.
Hunziker et al. [20], 2011	Switzerland	Simulation study	108 teams	Effective leadership and teamwork improved CPR performance metrics.
Weigl et al. [26], 2012	Germany	Prospective observational study	36 physicians	Workflow interruptions increased workload and impaired performance.
Peberdy et al. [31], 2008	USA	Registry analysis	86,748 IHCA patients	Survival was lower during nights and weekends, suggesting staffing-related disparities.
Hallstrom et al. [36], 2006	USA	Randomized trial	1,071 OHCA patients	Mechanical CPR showed no survival advantage over manual CPR.
Huis In 't Veld et al. [39], 2017	USA	Observational study	23 cardiac arrest cases	POCUS use during CPR caused prolonged interruptions in chest compressions.
Dudek et al. [43], 2023	Poland	Systematic review/meta-analysis	3,265 patients	POCUS findings correlated with resuscitation outcomes, but evidence remained heterogeneous.
Dudley et al. [47], 2009	USA	Observational study	50 trauma resuscitations	Family presence did not reduce resuscitation efficiency in pediatric trauma.
Afzali Rubin et al. [49], 2023	International	Cochrane systematic review	Multiple studies	Family presence during resuscitation improved family satisfaction without harming care quality.
Nolan et al. [44], 2014	UK	National audit analysis	22,628 IHCA patients	National audit systems improved benchmarking and identified survival variability.

System-level challenges

System-level operational factors within the ED substantially influence the quality of cardiopulmonary resuscitation and survival following IHCA. Persistent inter-hospital variation in survival rates, even after adjustment for patient case mix, indicates that structural and institutional processes contribute significantly to resuscitation outcomes [1,3]. These findings suggest that successful cardiac arrest management depends not only on the technical competence of individual providers but also on the efficiency of the surrounding resuscitation system.

ED crowding is one of the most significant operational barriers affecting CPR quality. Overcrowding has been associated with delays in defibrillation, with one study reporting a median delay of 42 seconds (IQR 22-78 seconds) alongside an 11% reduction in CCF (OR 1.4, 95% CI 1.1-1.8) [9]. Since both early defibrillation and maintenance of a high CCF are critical determinants of successful resuscitation, these delays directly compromise CPR effectiveness. Similarly, increased patient-to-nurse ratios adversely affect resuscitation timeliness; ratios exceeding 4:1 were associated with an increase in time-to-CPR initiation to 91 seconds (p < 0.01) [31]. These results indicate that staffing problems can directly lead to worse CPR performance metrics.

Institutional preparedness and team organization further influence the quality of resuscitation efforts. Hospitals without pre-designated resuscitation teams have demonstrated lower CCF values (78% vs. 89%) and a 2.1-fold increase in defibrillation delays compared with institutions using organized code team models [20]. In addition, delays in accessing essential resuscitation equipment, such as crash carts, have been reported with a median delay of 37 seconds [5]. These interruptions in workflow may reduce adherence to guideline-recommended CPR benchmarks and delay life-saving interventions.

Another major contributor to reduced resuscitation quality is the failure to identify and escalate physiological deterioration before cardiac arrest occurs. Approximately 80% of IHCAs are preceded by abnormal vital signs, yet institutions lacking electronic early warning triggers have shown reduced survival outcomes (OR 0.81) [6]. Delayed recognition of deterioration can postpone team activation, increasing no-flow time and lowering CPR quality once arrest occurs. This highlights the importance of robust monitoring and escalation systems in preserving arrest preparedness.

These system-level differences likely explain much of the observed inter-hospital variation in cardiac arrest outcomes. Hospitals with monitored settings demonstrate improved return of spontaneous circulation (ROSC) rates compared with unmonitored areas (OR 1.6) [4]. Access to defibrillation within two minutes improves survival in shockable rhythms by 2.3-fold [1]. Also, shorter response times are linked to better CPR quality. For example, a response time of less than three minutes results in a CCF of 85%, while a response time of six minutes results in a CCF of only 71% [3]. Together, these findings provide strong evidence that operational efficiency directly affects measurable CPR quality indicators and survival outcomes.

Overall, system strain in the ED impacts key components of high-quality resuscitation, including CCF, time-to-defibrillation, and response intervals. Strengthening staffing models, ensuring rapid access to resuscitation equipment, implementing organized code team activation, and improving early warning systems may enhance CPR quality and reduce variability in cardiac arrest outcomes across institutions.

Team and human-factor challenges

High-quality cardiopulmonary resuscitation depends not only on technical skill but also on effective team coordination, leadership, and cognitive performance under extreme time pressure. During cardiac arrest resuscitation, designated team leadership plays a critical role in coordinating role allocation, maintaining situational awareness, and prioritizing interventions, all of which are essential for minimizing interruptions in chest compressions and ensuring adherence to resuscitation protocols [21,26] In addition, psychologically safe team environments facilitate open communication, early error recognition, and rapid correction of performance deviations, thereby improving overall resuscitation quality [23].

Airway management is one of the hardest human-factor problems to address during resuscitation. It is also one of the largest sources of cognitive burden and a drop in the quality of CPR. Securing an advanced airway requires simultaneous coordination of ventilation, rhythm assessment, and defibrillation readiness, increasing the cognitive demands placed on the team leader. Studies have shown that endotracheal intubation may require pauses of 15-25 seconds, reducing CCF from 85% to 68% [32,33]. This interruption compromises coronary and cerebral perfusion and may adversely affect the likelihood of ROSC. Furthermore, airway management has been associated with a 42% increase in team leader cognitive load due to the need to manage competing priorities during a highly time-sensitive event [33]. Although supraglottic airway devices may reduce interruptions and restore CCF more rapidly, they remain susceptible to malposition and displacement, limiting their effectiveness in some scenarios [34].

Simulation-based studies further demonstrate the impact of airway tasks on team performance. Compared with compression-only resuscitation scenarios, the addition of airway interventions has been associated with a threefold increase in leadership errors and increased deviations in CPR performance, including a 25% increase in compression rate variability [21]. These findings suggest that airway interventions substantially increase cognitive workload, impairing leadership performance and CPR quality even in controlled environments.

Real-world observational studies confirm that these human-factor challenges translate into clinically relevant delays during actual resuscitations. The absence of clear leadership has been associated with a 2.1-fold increase in defibrillation delays, while endotracheal intubation during active resuscitation has been shown to double pause duration compared with standard rhythm checks (18 seconds vs. nine seconds) [20,26,32]. These observations indicate that airway management and leadership challenges significantly affect resuscitation flow under real clinical conditions, where environmental distractions and competing tasks further compound cognitive burden.

Acute stress during cardiac arrest management also impairs clinical decision-making and increases the likelihood of errors. Under high-stress conditions, clinical error rates have been reported to increase by 32%, while cognitive biases, such as anchoring and premature closure, may negatively influence rhythm recognition and treatment decisions [24,25]. In the ED, workflow interruptions, simultaneous critical cases, and environmental noise can increase team cognitive workload by 28%, further reducing the ability to maintain optimal CPR performance [26].

Provider fatigue represents another important human-factor barrier to effective resuscitation. Overnight shifts and prolonged working hours have been associated with a 19% reduction in adherence to CPR guidelines, potentially affecting both technical performance and team coordination [31]. Conversely, structured post-event debriefing has been shown to improve subsequent CCF by 12% and ROSC rates by 8%, suggesting that reflective team learning may mitigate some of these performance deficits [32,33].

Taken together, these findings demonstrate that CPR quality is shaped not only by technical proficiency but also by human factors such as leadership, cognitive workload, stress, and fatigue. Airway management, in particular, represents a major cognitive bottleneck that can interrupt compressions and impair team coordination. Addressing these human-factor challenges through structured leadership models, simulation-based training, and debriefing processes may improve CPR quality and resuscitation outcomes.

Technology and monitoring-related challenges

Technological adjuncts and advanced monitoring tools have been increasingly incorporated into cardiac arrest management to improve adherence to resuscitation guidelines and optimize clinical decision-making. However, despite their theoretical advantages, the impact of these technologies on CPR quality metrics and survival outcomes remains variable, often limited by workflow barriers, delayed implementation, and inadequate provider training.

Real-time CPR feedback devices provide immediate auditory and visual guidance regarding compression depth, rate, recoil, and CCF, thereby improving adherence to guideline-recommended targets such as compression depth of 5-6 cm, rate of 100-120 compressions per minute, and CCF greater than 80% [13-15,17,18]. Randomized trials have demonstrated measurable improvements in CPR quality, including increases in CCF from 78% to 92% and reductions in peri-shock pauses from 24 seconds to 11 seconds [19]. These improvements indicate that feedback devices can enhance technical CPR performance. However, evidence demonstrating a corresponding improvement in survival outcomes remains inconsistent. Furthermore, the constant flow of feedback data may increase the cognitive workload for resuscitation leaders. This finding shows that the usefulness of these devices depends not only on their availability but also on how well the team is trained and how well they fit into the resuscitation process [26].

ETCO₂ monitoring offers continuous real-time assessment of pulmonary blood flow during CPR and is recommended as a key physiological marker of resuscitation quality, with values greater than 10 mmHg associated with a higher likelihood of ROSC [16]. Waveform capnography is also useful for telling the difference between real ROSC and pseudo-electromechanical dissociation, with a reported sensitivity of almost 100% [19]. Accordingly, the AHA 2025 guidelines recommend quantitative waveform capnography as a class I intervention during cardiac arrest [11]. Despite this recommendation, early ETCO₂ implementation in the ED is often delayed by 20-30 seconds due to setup requirements, limiting its ability to guide interventions during the most critical early phase of resuscitation [16]. Thus, while ETCO₂ improves monitoring precision, its effectiveness depends on rapid and reliable implementation.

Mechanical chest compression devices, such as LUCAS Chest Compression System (Jolife AB, Lund, Sweden) and AutoPulse (ZOLL Medical Corporation, Chelmsford, MA), are designed to deliver consistent, fatigue-free compressions and have demonstrated improved CPR quality metrics, particularly in prolonged resuscitation or during patient transport. Studies have shown higher CCF values with mechanical devices compared to manual CPR (92% vs. 78%) [35,36]. However, despite improving compression consistency, three randomized controlled trials found no significant survival advantage compared with manual compressions (OR 1.01, 95% CI 0.87-1.18) [37]. Furthermore, setup times of 25-45 seconds and inadequate familiarity with the devices may interrupt compressions and offset their theoretical benefits. These results indicate that enhancements in compression quality do not inherently lead to better clinical outcomes without effective implementation.

Point-of-care ultrasound (POCUS) has become an important adjunct during cardiac arrest management, particularly for identifying reversible causes such as cardiac tamponade, massive pulmonary embolism, and hypovolemia, as well as for detecting organized cardiac activity. In terms of CPR quality metrics, however, POCUS use may negatively affect chest compression continuity. Studies report that ultrasound assessments during rhythm checks prolong pauses to 12-19 seconds per view, exceeding the recommended 10-second limit and reducing CCF by 8-12% [38,39]. Regarding survival outcomes, current evidence does not demonstrate a clear survival benefit from routine POCUS use during resuscitation, with no significant improvement in hospital discharge rates reported across studies (RR 0.44, 95% CI 0.22-0.88; OR 1.1, 95% CI 0.8-1.5) [40-43]. Nevertheless, POCUS improves prognostic accuracy, with superior sensitivity and specificity for detecting cardiac activity compared with manual pulse checks (100% and 98%, respectively, vs. 80% and 91%) [40]. Additionally, cardiac standstill on ultrasound predicts failure of ROSC in approximately 96% of cases, while organized cardiac activity may support continued resuscitative efforts. Recognizing both the benefits and risks, the AHA recommends a “pause-for-purpose” approach, limiting ultrasound imaging to less than 10 seconds during rhythm analysis [11]. Therefore, while POCUS enhances diagnostic assessment, its impact on outcomes depends heavily on operator skill and minimizing interruptions in compressions.

Overall, these technologies can improve selected CPR quality metrics and enhance clinical assessment during cardiac arrest, but researchers have not uniformly demonstrated consistent survival benefits. Implementation barriers, including delayed setup, limited device availability, inadequate training, and workflow misalignment, often reduce their effectiveness in real-world ED settings [30]. These findings emphasize that technology alone is insufficient; its successful impact on resuscitation outcomes depends on integration into coordinated team-based resuscitation systems.

Patient-related challenges

Patient-specific physiological and clinical characteristics substantially influence both immediate resuscitation success and long-term neurological outcomes, even when guideline-directed cardiopulmonary resuscitation is delivered. Initial arrest rhythm, metabolic status, physiological reserve, and the reversibility of underlying causes are key determinants of whether adequate CPR mechanics translate into ROSC and survival [28].

Among these factors, the initial arrest rhythm remains one of the strongest predictors of outcome. Patients presenting with shockable rhythms such as ventricular fibrillation and pulseless ventricular tachycardia have survival rates three to five times higher than those with non-shockable rhythms, such as pulseless electrical activity and asystole, largely because of the effectiveness of defibrillation in restoring organized cardiac activity [44]. This highlights that identical CPR quality metrics may yield markedly different outcomes depending on the underlying electrophysiological mechanism of arrest.

Metabolic derangements frequently encountered in critically ill ED patients can significantly reduce responsiveness to resuscitative interventions. Severe acidosis, hyperkalemia, and hypovolemia may make the myocardium resistant to both defibrillation and vasoactive drugs, which makes chest compressions less effective even when they are done correctly. The AHA's current guidelines stress the need to quickly find and fix reversible causes of cardiac arrest. These are often grouped together as the “5 Hs and 5 Ts”: hypovolemia, hypoxia, hydrogen ion (acidosis), hypo-/hyperkalemia, hypothermia, tension pneumothorax, cardiac tamponade, toxins, pulmonary thrombosis, and coronary thrombosis [11]. Failure to recognize and treat these reversible factors may delay ROSC despite optimal CPR delivery.

Another important factor in successful resuscitation is quick access to blood vessels, but achieving this can be hard to do in people who have peripheral edema, are very dehydrated, or have a history of using intravenous drugs. In such situations, delays in obtaining peripheral intravenous access may postpone administration of epinephrine and antiarrhythmic medications by several minutes, reducing the effectiveness of advanced cardiac life support interventions. Intraosseous access provides a faster and more reliable alternative, with reported success rates of 92% compared with 59% for intravenous access at 90 seconds, and is recommended by the AHA as a class I intervention when intravenous access cannot be rapidly established during cardiac arrest [11,45]. Early use of intraosseous access may minimize interruptions in resuscitation workflow and support timely medication delivery.

Patient age and pre-arrest physiological reserve also influence outcomes following cardiac arrest. Older adults, particularly those above 75 years of age, often exhibit reduced physiological resilience and a higher burden of comorbidities, which may limit the likelihood of ROSC and survival, despite guideline-compliant CPR. Pre-arrest instability, including hypotension, tachypnea, and hypoxia, has been shown to independently triple mortality risk [6]. These findings suggest that CPR quality and the patient’s underlying physiological capacity to recover both influence the effectiveness of resuscitation.

Mechanical factors related to the patient care environment may further affect the efficacy of chest compressions. Studies have indicated that chest compressions delivered on soft mattress surfaces can reduce effective compression depth by 20-30% unless a rigid backboard is used, potentially compromising cardiac output despite apparently adequate compression feedback [46]. To solve this problem, the AHA suggests using a firm surface or backboard to maintain a target compression depth of 5-6 cm during resuscitation [11]. This illustrates that environmental and patient-interface factors may influence CPR effectiveness even when compression metrics appear satisfactory.

Taken together, these findings demonstrate that CPR quality metrics must be interpreted within the context of patient-specific physiological conditions. Adequate compression depth, rate, and CCF do not guarantee successful resuscitation when severe metabolic abnormalities, delayed vascular access, advanced frailty, or reversible untreated causes are present. Recognition of these patient-related challenges is essential for understanding variability in cardiac arrest outcomes and for tailoring resuscitation strategies to individual clinical circumstances.

Ethical, legal, and documentation challenges

In the ED, healthcare providers often handle cardiac arrest with scant historical data and ambiguous patient preferences. Decisions about starting or stopping resuscitation need to be carefully weighed against beneficence, non-maleficence, and respect for autonomy [29].

Family presence during resuscitation (FPDR) adds operational and psychological complexity. Operationally, family witnessing increases team stress (self-reported 28% higher cognitive load) and pause duration during rhythm checks (+4.7 seconds average), though total CPR quality metrics remain equivalent [47,48]. Psychologically, 85-90% of families report reduced anxiety and improved closure with FPDR, but 15-20% of staff experience moral distress or second-guess decisions [49].

ED-specific protocol

A designated family liaison (not a team member) handles communication, which cuts down on the mental workload of clinical staff by 35%. AHA 2025 supports FPDR with a trained liaison when family requests presence [11]. Unstructured FPDR risks interruptions (e.g., door opening, questions during compressions), emphasizing the need for an institutional policy. Documentation during resuscitation events is often incomplete or inconsistent. Failure to accurately record compression fraction, defibrillation timing, or clinical decision-making limits opportunities for quality improvement and structured debriefing. Precise documentation is essential for both clinical governance and medico-legal accountability. Global disparities in access to monitoring technologies and critical care infrastructure further affect the fair implementation of evidence-based resuscitation practices [30]. These disparities require us to interpret resuscitation science within the constraints of differing health system capacities.

Integrated perspective

**Figure 1:**
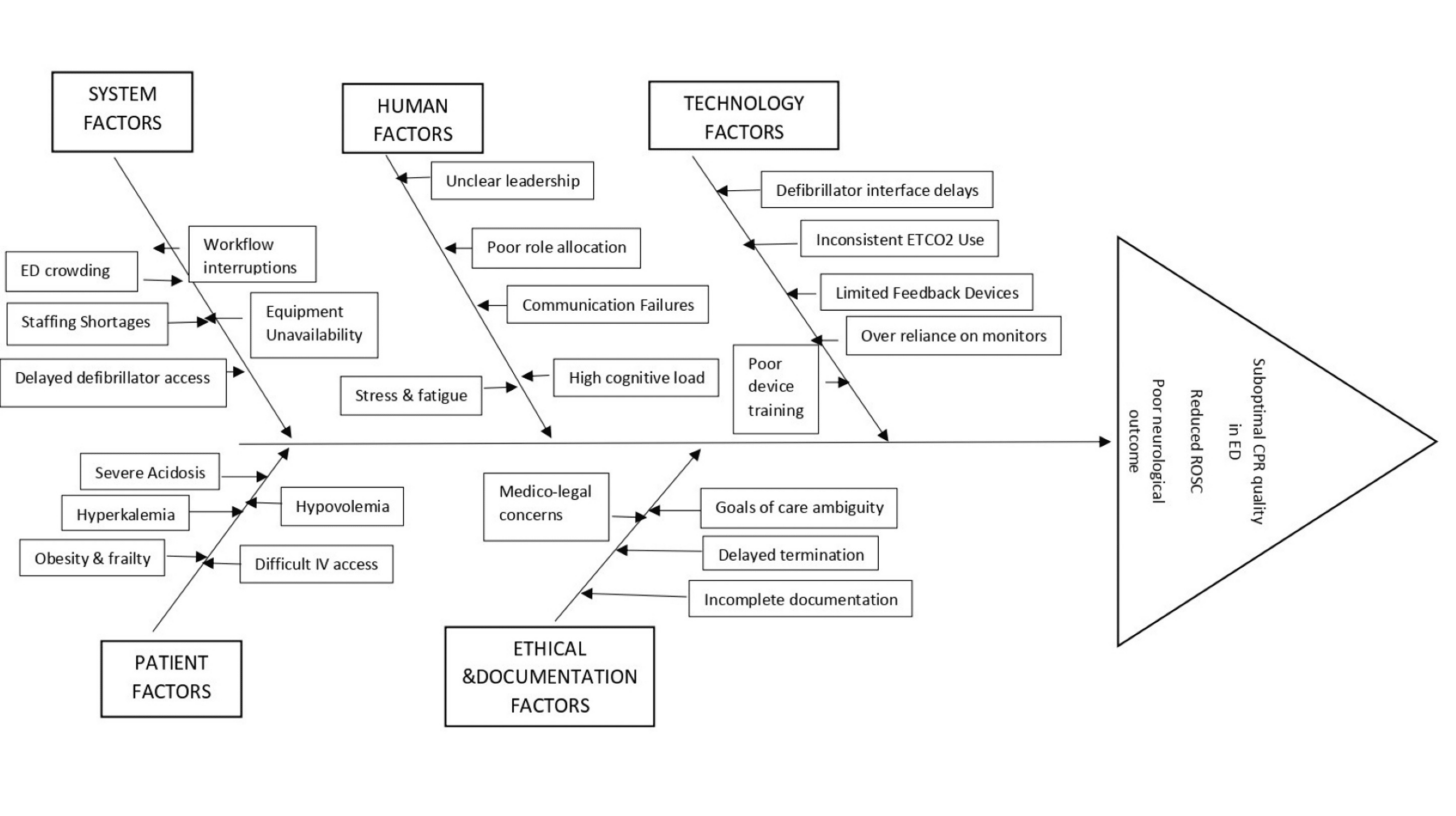
Root causes influencing CPR quality in the emergency department: a fishbone framework This fishbone diagram shows the various factors that affect how well CPR works in the emergency department, including system demands, team interactions, human factors, technology use, patient complexity, and ethical or documentation issues. These interacting domains may affect performance variability and impact survival outcomes.

The determinants described above can be conceptualized as interacting contributors to CPR performance variability (Figure 1). International guidelines clearly define technical targets for CPR performance, including compression depth and rate, recoil, defibrillation timing, and physiological monitoring [50-52]. However, consistent implementation in ED settings remains variable.

Extensive registry analyses have recorded variations in survival and epidemiological trends [1-3]. Whereas CPR quality metrics have illustrated correlations with outcomes [13-15]. Human factors research emphasizes leadership and stress-related performance degradation [20,21,24], and technological studies examine ETCO₂ monitoring and feedback systems [16-19]. Yet these domains are often evaluated independently.

In practice, the ED serves as a setting where system strain, cognitive load, technological variability, patient complexity, and ethical decision-making occur simultaneously. 

In many EDs, competing priorities, such as simultaneous trauma activations or limited staffing, may make strict protocol adherence difficult despite best intentions. Table 2 presents a summary of the multidimensional determinants that influence CPR quality in the ED.

**Table 2:** Multidimensional determinants of CPR quality in the emergency department DNR - do not resuscitate; ETCO₂ - end-tidal carbon dioxide; ROSC - return of spontaneous circulation

Domain	Key Determinants	Mechanism Affecting CPR Quality	Representative Evidence
System-Level Factors	ED overcrowding, staffing ratios, workflow interruptions, delayed defibrillator access, absence of standardized arrest protocols	Reduced chest compression fraction, delays in defibrillation, fragmented coordination, increased cognitive overload	Registry analyses and ED-based observational studies demonstrating association between system strain and survival variability
Human Factors	Leadership clarity, teamwork dynamics, communication failures, cognitive load, psychological safety, fatigue	Inconsistent compression depth/rate, prolonged pauses, delayed rhythm recognition, medication timing errors	Simulation-based studies and human factors research linking team performance to CPR metrics
Technological Influences	Real-time CPR feedback devices, capnography (ETCO₂) monitoring, mechanical compression devices, defibrillator interface usability	Improved compression depth/rate adherence, enhanced monitoring of perfusion, reduced variability in performance	Interventional quality improvement trials and guideline recommendations
Patient-Related Characteristics	Age, comorbidities, initial rhythm, body habitus, pre-arrest physiology, metabolic derangements	Altered physiologic response to CPR, difficulty achieving adequate compression depth, variable ROSC likelihood	Cohort studies evaluating predictors of survival and neurologic outcome
Ethical and Legal Dimensions	DNR ambiguity, goals-of-care uncertainty, termination-of-resuscitation decisions, documentation practices	Delayed decision-making, prolonged non-beneficial resuscitation, moral distress affecting team performance	Guideline statements and ethical analyses in emergency medicine literature

Translational implications

One of the most effective ways to translate guideline theory into practice is the formal designation of a CPR coach. This individual is not the team leader; instead, they focus exclusively on the physics of the code. By standing at the foot of the bed and providing a human interface for ETCO₂ data and metronome pacing, the coach ensures that compression quality does not degrade as the team leader focuses on diagnostic uncertainty.

Traditional training in a lab is beneficial, but it doesn't account for the physical constraints of a crowded ED. Conducting unannounced mock codes in the actual resuscitation bays allows teams to identify latent safety threats. This process includes finding where the backboard is stored, testing the reach of the defibrillator cables, and managing the physical flow of personnel in a tight space.

Improving cardiac arrest outcomes in the ED requires coordinated, multifaceted strategies rather than isolated training efforts. Structured leadership frameworks, standardized documentation tools, real-time feedback technologies, and institutional quality improvement initiatives may collectively enhance adherence to evidence-based recommendations and reduce performance variability.

Implementation must also consider local resource availability, particularly in settings with limited access to advanced monitoring or staffing support [30]. Adaptation to local resource conditions is essential for sustained improvement.

Limitations

This review has limitations inherent to its narrative design. Although a structured search strategy was used and screening was performed systematically, formal quality appraisal tools were not applied. The synthesis represents interpretive integration rather than quantitative meta-analysis. Heterogeneity across study designs limits direct comparison, and the predominance of studies from high-income healthcare systems may restrict generalizability to resource-limited settings.

However, the narrative format allows integration across systems science, human factors, technology, and ethics areas that are not easily combined through quantitative pooling methods.

## Conclusions

Accomplishment of consistent high-quality CPR in the ED is determined by far more factors than technical skills alone. Survival outcomes may depend on operational pressure, quality of the team, work and coordination, monitoring technology, real-time decision-making, and patient-dependent factors. Persistent survival variability across institutions reflects differences in organizational structure and clinical environment. Enhancing the outcomes necessitates a system-based approach encompassing structured leadership development, simulation-based team training, real-time performance feedback, standardized documentation, and workflow optimization.

Future research should focus on how resuscitation best practices can be reliably applied across different clinical settings, making sure that advances in care lead to better survival outcomes for all patients.
